# The Relationship Between Caffeine and Caffeinated Drinks in Causing Intracranial Hemorrhage in the Elderly Aspirin-Taking Population: A Systematic Review

**DOI:** 10.7759/cureus.17783

**Published:** 2021-09-06

**Authors:** Aafreen Khan, Mohammed A Abdalla, Christine M Zakhary, Hiam Rushdi, Jaafar A Hamdan, Kerolos N Youssef, Safeera Khan

**Affiliations:** 1 Internal Medicine, California Institute of Behavioral Neurosciences & Psychology, Fairfield, USA; 2 Family Medicine, California Institute of Behavioral Neurosciences & Psychology, Fairfield, USA

**Keywords:** acetylsalicylic acid, caffeine, aspirin, intracranial hemorrhage, non-steroidal anti-inflammatory drug

## Abstract

Caffeinated drinks are the most widely consumed beverages globally and their intake has increased in the elderly. Caffeine exhibits dose-dependent adverse effects. Low to moderate doses cause anxiety, restlessness, irritability, and nausea. High doses of 3-5g can affect different physiological systems and lead to detrimental effects like palpitations, hypertension, agitation, seizures, and coma. Low-dose aspirin is the most used anticoagulant in preventing ischemic vascular events. An increased risk of intracranial hemorrhage is associated with low-dose aspirin with an intensified intracerebral hemorrhage risk. The aim of this research is to explore the association between caffeine and aspirin in causing lethal intracranial hemorrhage in the older population. Because of the devastating nature of intracranial hemorrhages and the inconsistent published data on the risk of intracranial hemorrhage in individuals taking both aspirin and caffeine, we conducted a systematic review considering the elderly population.

We conducted the study following the reporting guidelines for systematic review and the Preferred Reporting Items for Systematic Reviews and Meta-analyses (PRISMA) checklist. Inclusion and exclusion criteria were determined. Data was collected from PubMed, PubMed Central® (PMC), National Library of Medicine (MEDLINE), Google Scholar, Multidisciplinary Digital Publishing Institute (MDPI), and Web of Science by applying keywords and Medical Subject Headings (MeSH) terms individually. Our initial search yielded 155,270 articles, which were scrutinized, and duplicates were removed for accuracy. Of these, a total of 13 research papers were finally extracted using the PRISMA recommendations and applying other inclusion and exclusion criteria. With the help of our systematic review, we could determine that both aspirin and caffeine portrayed a role in causing intracranial hemorrhage independently, but further studies are recommended to evaluate if both could lead to similar adverse effects when taken collectively.

## Introduction and background

“AM: Coffee… PM: Still coffee”

**Figure 1:**
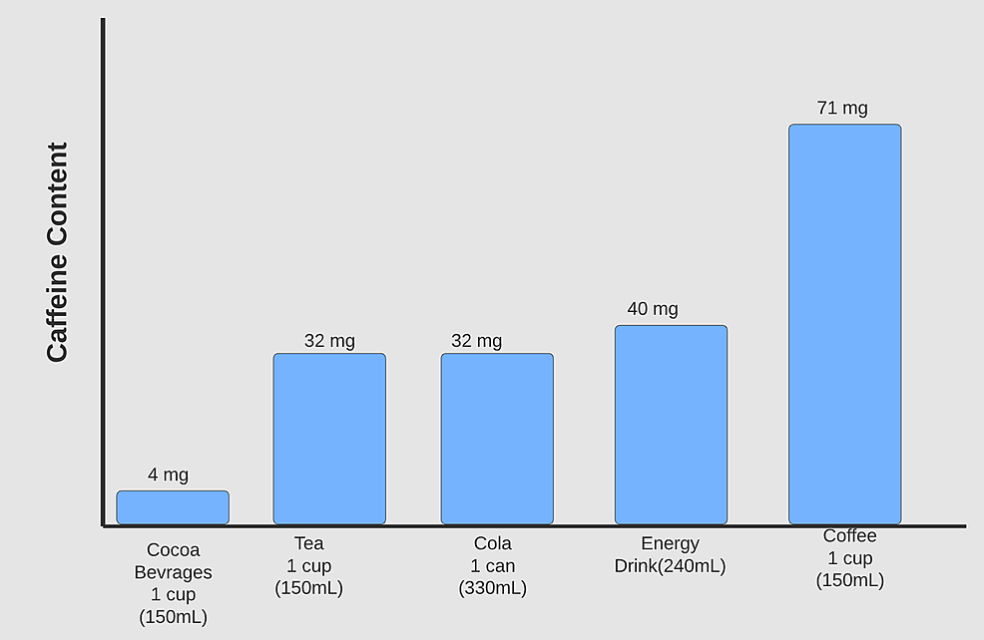
The minimum caffeine content in common foods and beverages.

Caffeine is the most consumed central nervous system stimulant in the world [1]. It is a component of various dietary sources, including coffee, tea, candy bars, cocoa beverages, energy drinks, and soft drinks. The caffeine content of these dietary sources differs as follows: coffee: 71-220 mg per 150 mL, tea: 32-42 mg per 150 mL, cola drinks: 32-70 mg per 330 mL, and cocoa beverages: 4 mg per 150 mg [2]. It is anticipated that more than 80% of the world’s population and up to 89% of the United States (US) population consumes caffeine. The average daily consumption of caffeine varies and is registered to be 142 mg each day for adults and children in the US, a decline from the previous years [3]. Consumption surveys performed by the coffee industry suggest that 64% of the US population aged 18 years and older drink a cup of coffee every day. According to the National Health and Nutritional Examination Survey (NHANES), coffee consumption is higher in the older age groups, with 72% of adults over 60 years being considered coffee drinkers [4]. Figure 1 shows caffeine content in different beverages.

The pharmacological effects of caffeine on the central nervous system usually occur at 15 mg/L or higher [5]. Caffeine exhibits dose-dependent effects with desirable effects at lower doses (i.e., ≤ 400 mg) and detrimental effects above this level, although there is considerable inter-individual variation. For example, at doses of 250 mg, increased arousal, alertness, concentration, and well-being have been noted in human subjects. In contrast, increased tension, nervousness, anxiety, excitement, irritability, nausea, paraesthesia, tremor, perspiration, palpitations, restlessness, and possibly dizziness occur at a dose of 500 mg. Sub-lethal doses of 7-10 mg/kg produce symptoms such as nausea, headache, chills, flushing, palpitations, and tremor, although individuals’ responses may vary significantly [3].

**Figure 2:**
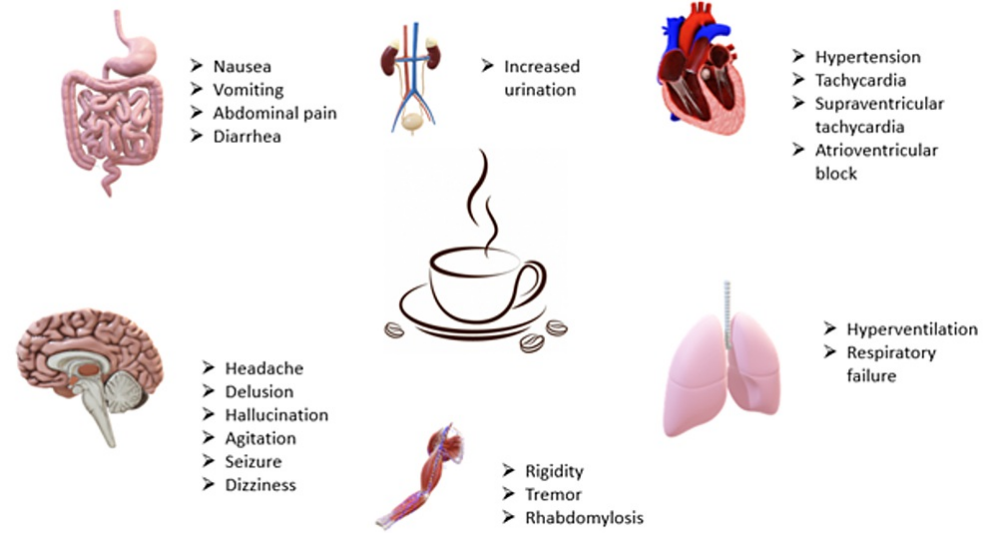
Clinical features of caffeine intoxication affecting various systems

Toxic symptoms begin to manifest at 1 g and a dose of 2 g requires hospitalization. Higher doses, typically 5g or more, could be lethal. However, some have determined that lower doses of about 3 g could also be deadly under certain circumstances. The clinical features of caffeine intoxication differ and are known to cause gastrointestinal symptoms like nausea, vomiting, abdominal pain, diarrhea, cardiovascular symptoms such as hypertension, bradycardia, hypotension, tachycardia, atrioventricular block, supraventricular tachycardia (SVT), and neuropsychological symptoms, which include delusions, hallucinations, anxiety, excitation, agitation, seizures, headache, cerebral edema, and coma. Hyperventilation, respiratory failure, weakness, rigidity, tremor, rhabdomyolysis, tinnitus, dizziness, diuresis, and death are also known to occur [3]. Figure 2 shows the effects of caffeine on various systems of the body.

Caffeine also causes chronic toxicity, including hypokalemia, anorexia, nausea, vomiting, palpitations, dysrhythmia, seizures, and a constellation of symptoms referred to as 'caffeinism', which typically occurs with daily intakes of 1 to 1.5 g [3]. These physiological and behavioral symptoms of caffeine occur predominantly through the antagonism of the endogenous adenosine A1 and A2A receptors [6]. Caffeine is also known to increase blood pressure when associated with stressful events. In addition, an association between the use of caffeinated pharmaceutical products and intracerebral hemorrhage was established [7].

Intracranial hemorrhagic events are generally associated with higher mortality and more significant disability than ischemic events related to atherosclerotic cardiovascular disease such as ischemic stroke. Low-dose aspirin is the most widely used agent for preventing cardiovascular disease in the elderly population. Low-dose aspirin used in the primary prevention of cardiovascular events without symptomatic cardiovascular disease was associated with an increased risk for intracranial hemorrhage, particularly in Asian populations in patients with lower BMI [8]. Of the various major bleeding events related to the use of aspirin, intracranial hemorrhage is a particular concern as it is firmly associated with a high risk of mortality.

Although there are complete reviews published on caffeine, there has been minimal detailed research regarding caffeine fatalities and their effects on intracranial hemorrhage if combined with aspirin in adults. The older population is subjected to increased strokes due to atherosclerotic changes and various other risk factors. With the use of caffeine and aspirin on the rise, we recognized the importance of conducting a systematic review to know if caffeine and aspirin could be a leading cause of Intracranial hemorrhage in the elderly aspirin-taking population. 

## Review

Protocol

We conducted a systematic review following the PRISMA guidelines [9].

Inclusion/exclusion criteria

We selected studies from the past nine years, from 2012 to 2021, that independently dealt with the aspirin-taking elderly population in whom caffeine intake is associated with intracranial hemorrhage. Full-text studies done in humans were included, excluding all those in animals. The studies included were in the English language. Only the older aspirin-taking population studies were considered. Finally, grey literature, unpublished literature, and studies beyond nine years were excluded. 

Data source and search strategy

We searched for articles indexed between 2012 and 2021 in PubMed, PubMed Central® (PMC), National Library of Medicine (MEDLINE), Google Scholar, Multidisciplinary Digital Publishing Institute (MDPI), and Web of Science from April 10 to April 14, 2021. We applied keywords and MeSH terms individually to detect relevant articles. At the end of our search, we eliminated duplicate articles. Table 1 and Table 2 show the search results.

**Table 1:** Database search results using regular keywords

Keywords	Database used	Results before Inclusion/exclusion criteria	Results after Inclusion/exclusion criteria	Results after screening
Caffeine and intracranial hemorrhage	PubMed	42	11	2
Caffeine and aspirin	PubMed	13	2	1
Aspirin and intracranial hemorrhage	PubMed	1308	163	13

**Table 2:** Database search using MeSH strategy

MeSH Strategy	Database Used	Results before Inclusion/exclusion criteria	Results after Inclusion/exclusion criteria	Results after screening
"Caffeine/poisoning"[Mesh] OR "Caffeine/toxicity"[Mesh] AND Intracranial hemorrhage OR Intraventricular bleeds	PubMed	8513	112	5
"Caffeine/adverse effects"[Mesh] OR "Caffeine/blood"[Mesh] OR "Caffeine/cerebrospinal fluid"[Mesh] "Aspirin/administration and dosage"[Mesh] OR "Aspirin/adverse effects"[Mesh]	PubMed	10,503	282	6
"Intracranial Hemorrhages/physiopathology"[Mesh] AND Aspirin OR acetylsalicylic acid OR blood thinner OR COX inhibitor OR anticoagulant OR "Aspirin/administration and dosage"[Mesh] OR "Aspirin/adverse effects"[Mesh]	PubMed	444,025	7,604	11

Data extraction

The titles, full-text articles, and abstracts were screened independently by two reviewers. The elements extracted from each study included the sample size, year of publication, study design, age range, and study outcome. Other reviewers also scrutinized the studies gathered by one reviewer for precision and eligibility. In case of disagreement, conflicts were resolved by a mutual discussion on the study in question.

Quality assessment

The tools used to assess the quality of the studies are shown in Table 3.

**Table 3:** Quality assessment using the preferred checklists SANRA: scale for the quality assessment of narrative review articles; JBI:  Joanna Briggs Institute; AMSTAR: assessment of multiple systematic reviews

TYPE OF STUDY	TOOL USED	NUMBER OF STUDIES
Case Reports	JBI checklist	1
Narrative review	SANRA checklist	1
Observational cohort studies	Newcastle-Ottawa tool	5
Randomized Controlled Trials	Cochrane risk-of-bias assessment tool	2
Case-control studies	Newcastle-Ottawa tool	4
Systematic Reviews	AMSTAR checklist	11

Results

Study Identification and Selection Results

**Figure 3:**
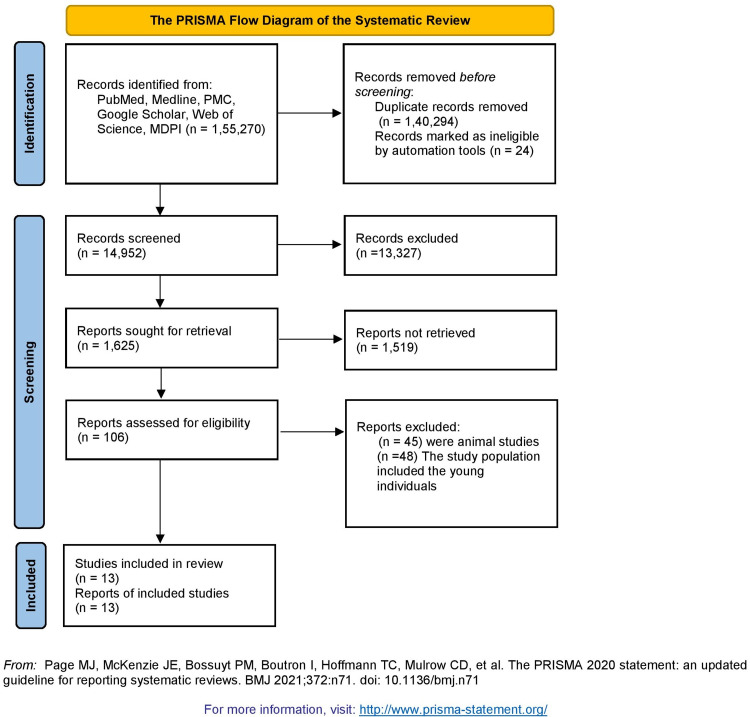
PRISMA flow diagram outlining the screening and selection process of articles obtained from different databases. PRISMA: Preferred Reporting Items for Systematic Reviews and Meta-Analyses

We used six databases to collect relevant articles: PubMed, PMC, MEDLINE, Google Scholar, MDPI, and Web of Science. Our initial search yielded 155,270 articles. Duplicates (n = 140,294) and ineligible records ( n = 24) were removed by automation tools and a total of 14,952 remained after the screening process. Abstracts and full-text articles were read thoroughly and inclusion criteria were applied. Finally, after a detailed quality assessment and exclusion of relevant articles, the final review included 13 articles. The search process in the form of a PRISMA flow diagram is depicted in Figure 3.

Discussion

Caffeine, a central nervous system stimulant, belongs to the class methylxanthine. It has numerous pharmacological and physiological effects on cardiovascular, respiratory, renal systems, and smooth muscles. It also affects mood, memory, alertness, and physical and cognitive performance. This review article will discuss the effects of caffeine on the brain and its potential role in causing intracranial hemorrhage when combined with aspirin in the elderly population.

Caffeine and its mechanism of action

Caffeine is rapidly absorbed from the gastrointestinal tract within 45 minutes of ingestion, 99% is completely absorbed. The functioning of the cardiovascular, respiratory, renal, and nervous systems is affected by absorption. The mechanisms of action of caffeine differ for different physiological effects, which are thought to be mediated through the following major mechanisms: Phosphodiesterase inhibition, the release of calcium from the intracellular stores, and adenosine receptor antagonism [10].

Caffeine and Phosphodiesterase Inhibition

Caffeine inhibits the enzyme phosphodiesterase in skeletal muscle and adipose tissues and increases the intracellular concentrations of cyclic adenosine monophosphate (cAMP), which leads to an increase in blood catecholamines. Caffeine is a weak phosphodiesterase inhibitor that accounts for cardio-stimulatory and antiasthmatic actions and is also effective as bronchiolar and tracheal relaxants [10].

Caffeine and Calcium Mobilization

In striated muscles, caffeine at high concentrations (0.5-1 mmol/L) impedes the uptake and storage of calcium by the sarcoplasmic reticulum and increases the translocation of Ca++ through the plasma membrane. It also increases the myofilament sensitivity to Ca++ by binding to ryanodine receptors in muscle and brain calcium channels. Even though caffeine has been shown to release calcium from intracellular storage pools, the threshold concentration required to observe this effect in vitro was substantially higher than the concentrations needed for cardiac stimulation in vivo. Therefore, this subcellular action of caffeine is physiologically irrelevant though it possibly could be relevant at toxic concentrations of caffeine [10].

Caffeine and Adenosine Receptor Antagonism

**Figure 4:**
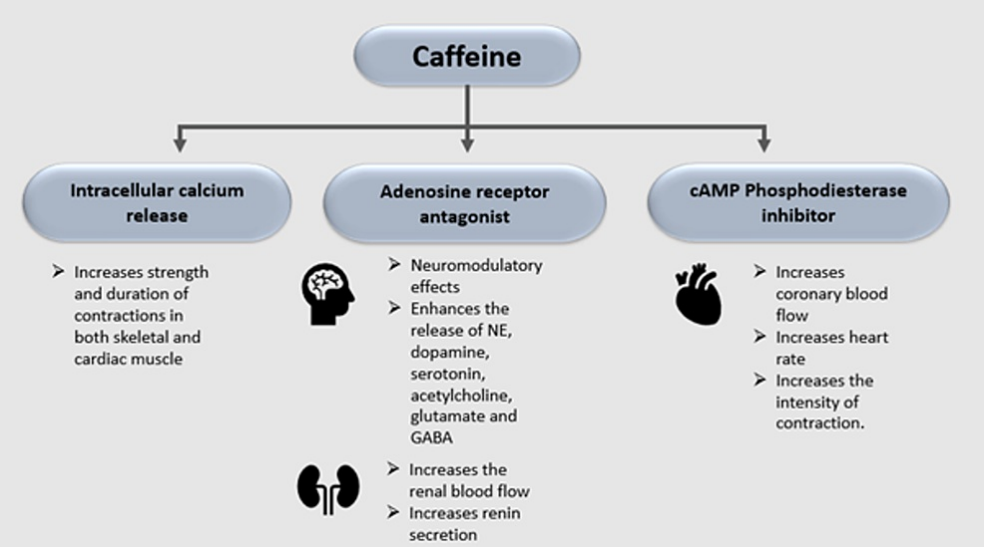
Physiological actions of caffeine cAMP: cyclic adenosine monophosphate; NE: norepinephrine; GABA: gamma-aminobutyric acid

Adenosine receptor blockade appears to be the predominant mode of action [2]. The ability of caffeine to inhibit adenosine receptors seems to be incredibly important in its effects on behavior and cognitive function. These effects are known to occur from the competitive binding of caffeine to the adenosine receptors. They are crucial in contributing to the effects on the central nervous system, especially the ones involved in the neuromodulator effects of adenosine. Due to the blockage of the inhibitory effects of adenosine through its receptors, caffeine indirectly affects the release of dopamine, norepinephrine, serotonin, acetylcholine, glutamate gamma-aminobutyric acid (GABA), and neuropeptides [10]. Figure 4 shows the physiological effects of caffeine.

Physiological effects of caffeine on brain

Caffeine, which acts as an antagonist at adenosine receptors, blocks the endogenous adenosine [11]. There are four types of adenosine receptors: A1, A2A, A2B, and A3. Caffeine at brain concentrations blocks the effects of the adenosine A1 and A2A receptors, with A2B participating only in pathological situations and A3 having a minimal affinity for caffeine [12]. The A1 adenosine receptors are widely distributed throughout the brain, within the elevated hippocampus, thalamus, cerebral and cerebellar cortex. On the contrary, the A2A receptors are exclusively located in the striatum, olfactory tubercle, and nucleus accumbens. In the latter regions, the A2A receptors are co-expressed with dopamine D2 receptors and enkephalin in the striatal neurons. There exists direct evidence of central functional interaction between dopamine D2 and adenosine A2 receptors. Administration of A2A receptor agonists decreases the affinity of dopamine D2 receptors in striatal membranes [2]. By removing the negative modulatory effects of adenosine at dopamine receptors, caffeine has been shown to stimulate dopaminergic activity. Studies suggest that the release of dopamine in the nucleus accumbens may be a specific neuropharmacological mechanism causing the addictive potential of caffeine. 

The behavioral stimulant effect of caffeine is due to adenosine antagonism. In addition to its direct effects on adenosine, increased locomotor activity is seen due to paraxanthine, a primary metabolite of caffeine [11]. Blood pressure surges considerably above baseline for older men following caffeine ingestion, whereas it remained statistically unchanged in the younger. Heart rates were unaffected in both groups on caffeine ingestion. The appearance and clearance rates of norepinephrine were unaffected by caffeine in both groups, although the older population had higher concentrations of norepinephrine with caffeine [13]. Several investigators have reported reducing cerebral blood flow (CBF) on intravenous administration of caffeine due to its cerebral vasoconstrictive effects by blocking adenosine which is a powerful vasodilator. Though the CBF reduction following caffeine administration does not appear severe enough to cause symptoms of cerebral ischemia in normal individuals, its risk in causing transient ischemic attacks and cerebral infarction in high-risk individuals and those recovering from cerebrovascular accidents is unclear [12].

Caffeine also exhibits psychopharmacological effects, including increased energy, increased alertness, improved mood, and enhanced cognitive performance. A dose-dependent decrease in heart rate and increase in blood pressure was shown in both males and females [12]. Chronic caffeine administration upregulates the adenosine system, which emerges as a neurochemical mechanism underlying caffeine withdrawal syndrome. This mechanism leads to augmented functional sensitivity to adenosine during the abstinence of caffeine and likely plays an important role in the behavioral and physiological effects produced by caffeine withdrawal [11].

Aspirin and its mechanism of action

Aspirin (acetylsalicylic acid) belongs to the non-steroid anti-inflammatory drug class. It has analgesic, antipyretic and anti-inflammatory effects; the last 50 years saw aspirin being used mainly in primary and secondary thromboembolic prevention as an antithrombotic agent [14]. Vane discovered that aspirin and other non-steroidal anti-inflammatory drugs (NSAIDs) exert anti-inflammatory, analgesic, and antipyretic actions by inhibiting the enzyme cyclooxygenase (COX) activity, which leads to the formation of prostaglandins (PGs) that cause inflammation, pain, swelling, and fever. The constitutive isoform COX-1 supports the beneficial homeostatic functions. In contrast, the inducible isoform COX-2 is upregulated by the inflammatory mediators, and its products cause symptoms of inflammatory diseases such as rheumatoid and osteoarthritis [15].

Aspirin inactivates COX-1 irreversibly and suppresses the production of prostaglandin H2, a precursor of thromboxane A2. This effect is achieved through the acetyl group of aspirin, which covalently attaches to Ser529 of the active site of the enzyme COX-1. Aspirin likewise interacts with the amino acid Arg120 and subsequently blocks the access of arachidonic acid to the hydrophobic channel to Tyr385 at the catalytic site, thus inhibiting the generation of prostaglandin H2. The antithrombotic effects of aspirin also include the acetylation of blood coagulation proteins, including fibrinogen. Therefore, aspirin promotes fibrinolysis. The inhibition of COX-2 by acetylating Ser516 is nearly 170-fold slower than the reaction with COX-1. Lei et al. stated the reaction with COX-2 to be 10 to 100 times slower than that with COX-1. Aspirin produces an irreversible defect in thromboxane synthesis for the lifetime of affected platelets, i.e., eight to 10 days. Aspirin causes downregulation of dense granule release in platelets. Only 10% of the platelet pool is replenished daily. Therefore, aspirin administration in low doses can entirely inhibit COX-1 producing long-lasting defects [16].

The mechanism of action of aspirin varies with the dose. At low doses, typically 75-81 mg/day, aspirin causes irreversible acetylation of serine 530 of COX-1. This inhibits the generation of thromboxane A2 by platelets, resulting in an antithrombotic effect. An Intermediate dose of 650 mg to 4 g/day of aspirin inhibits COX-1 and COX-2, blocking PG production, which exerts an analgesic and antipyretic effect. At a high dose of 4 to 8 g/day, it acts as a potent anti-inflammatory agent helpful in rheumatic disorders; the mechanism of action at such high doses may include both prostaglandin dependent (particularly COX-2-dependent prostaglandin E_2_ (PGE2) and independent effects [17].

Combined effects of aspirin and caffeine on brain

Normal aging is associated with significant alterations in the brain’s vascular structure and function, leading to increased strokes. Therefore, it is essential to address the potential risks of aspirin and caffeine in causing intracerebral hemorrhage in the elderly. The most common source of dietary caffeine in the elderly is coffee, with an average consumption of around 200 mg/day. Because of the more significant percentage of adipose tissue to lean body mass in adult humans, and because caffeine is distributed through lean body mass effectively, a dose of caffeine expressed as mg/kg total body weight may result in higher tissue and plasma concentrations in the elderly compared with younger individuals. The physiological and metabolic responses to caffeine are similar in the elderly and younger individuals. However, responses to caffeine in some physiological systems may be more significant in the elderly at doses in the range of 200 to 300 mg. Evidence also suggests that the sensitivity of the pressor effects of caffeine increases with age [18]. 

Caffeine has a considerable effect on blood pressure. A cross-sectional study by Garcia et al. conducted in 2016 assessed the relationship between chronic coffee consumption on 24-hour blood pressure control among hypertensive older adults. It included 1164 hypertensive individuals aged ≥63 years. The study results showed increased blood pressure among the 715 hypertensive participants who consumed ≥3 cups of coffee each day compared to non-coffee drinkers. A similar association was seen in smokers and individuals with excess body weight. Therefore, the studies concluded that habitual coffee consumption was associated with uncontrolled blood pressure in the older population [19].

Stressful events associated with caffeine and taurine can also increase blood pressure by triggering more adrenaline release, stimulating vasoconstriction. A significant association was found between the use of caffeine in pharmaceutical products and the risk for aneurysmal subarachnoid hemorrhage defining an association between intracerebral hemorrhage and caffeinated beverages. The pathophysiology of the reported aneurysmal rupture is thought to be caused by high blood pressure secondary to the abuse of caffeinated beverages. Therefore, it is helpful to be cautious and aware of caffeinated energy drinks and the potential health consequences of their use or abuse [7].

Elderly patients are comparatively at high risk for the development of vascular disease [20]. Low-dose aspirin is among the most widely used antiplatelet agents to prevent ischemic vascular events, from which a substantial benefit may also be expected on regular administration. Caution should be exercised on using low-dose aspirin as a primary prevention strategy in older individuals. It resulted in a significantly higher risk of significant hemorrhage and did not significantly lower the risk of cardiovascular disease [21]. Ando et al. conducted a cohort study to examine the influence of blood pressure on the efficacy of low-dose aspirin for the primary prevention of cardiovascular events. The study included individuals aged 60 to 85 years with dyslipidemia, hypertension, with or without diabetes, but with no history of cardiovascular disease. All participants received aspirin at a dose of 100 mg per day or no aspirin and were followed for a mean duration of about 5.02 years. The study resulted that aspirin had no significant impact on the primary outcome of death from cardiovascular disease, nonfatal stroke, and nonfatal myocardial infarction. There was no overall benefit of low-dose aspirin therapy, although it was associated with an elevated risk of hemorrhagic events [22]. Low-dose aspirin was associated with a slightly increased risk of intracranial bleeds in which the incidence of subdural hemorrhage (SDH) was higher in men.

In contrast, a high incidence of intracerebral hemorrhage (ICH) and subarachnoid hemorrhage (SAH) was seen in women. Aspirin was associated with a 34% increase in intracranial bleeds and a 55% increase in major bleeding [22]. Thus, if low-dose aspirin is given universally, adverse outcomes such as intracranial hemorrhage may outweigh the beneficial effects. Increased 90-day mortality was associated with the practice of anticoagulation treatment. The incidence of spontaneous first-time intracerebral hemorrhage was found in many patients who received anticoagulant therapy or used platelet inhibitors before admission. The mortality among these was higher than those who had received no such treatment [23]. 

In addition, few studies have addressed the association between caffeine and the risk of hemorrhagic stroke. Caffeine caused nearly identical blood pressure elevation in men and women, and women responded to caffeine with an increase in cardiac output. The interaction between caffeine and other elements within medications may also increase the risk of hemorrhagic stroke. Lee et al. performed a case-control study to evaluate the association between caffeine-containing medications and the risk of hemorrhagic stroke. A total of 2710 individuals with hemorrhagic stroke were screened for eligibility, out of which 940 patients with hemorrhagic stroke were matched to 1880 control subjects. The study included hospital and community-based control groups, each containing 940 individuals. Information on all the medications taken 14 days prior to the index date and time of stroke onset for case subjects was determined. The study resulted that caffeine in pharmaceutical products may increase the risk of hemorrhagic stroke, including both SAH and ICH. Even though these results are suggestive, further analysis should be performed to corroborate the association [1].

Although studies have shown that both caffeine and aspirin can lead to a significant risk of intracerebral bleeds independently, there is still a void that needs to be filled by conducting studies having a higher quality of evidence. Table 4 shows the summary of the studies included in the review.

**Table 4:** Summary of the selected studies included in the review.

Author	Year of publication	Type of the study	Purpose of study	Results/conclusion
Chawla et al. [2].	2015		Neurological effects of caffeine	
Meredith et al. [11].	2013	Review article	A comprehensive review on caffeine use disorder	Caffeine dependence leads to significant distress and functional impairment.
Ferre [12].	2014	Summary article	To examine the safety and efficacy of caffeine in food and dietary supplements.	Psychostimulatory effects of caffeine occur by blocking adenosine receptors.
Vane et al. [15].	2003	Review article	Mechanism of action of aspirin	Aspirin inhibits COX1, COX2 and exerts its antithrombotic, antipyretic, and analgesic effects
Mekaj et al. [16].	2015	Review article	To explore new insights into the mechanism of action of caffeine	Aspirin, a promising drug in preventing recurrent unprovoked venous thromboembolism.
Ambramson et al. [17].	2021		Mechanism of action and major toxicities of aspirin	Aspirin exhibits dose-dependent effects on COX1 and COX2. It also has anticoagulant, gastrointestinal adverse effects.
Massey [18].	2012	Review article	Effects of caffeine in the elderly.	Increasing age is associated with increased sensitivity to the pressor effects of caffeine.
Garcia et al. [23].	2016	Cohort study	Coffee consumption and its effects on blood pressure	Habitual coffee consumption was associated with uncontrolled blood pressure in the hypertensive older population.
Mahe’ et al. [20].	2003	Review article	To study aspirin and its role in preventing cardiovascular events in the Elderly.	Aspirin has a higher risk of bleeding in the elderly; therefore, it should be given as primary or secondary prevention only after a comprehensive evaluation.
Argano et al. [7].	2012	Case study	To establish a relationship between energy drink abuse, hypertension, and break-up of cerebral aneurysm.	Energy drink abuse led to the rupture of an aneurysm by causing high blood pressure.
Mc Neil et al. [21].	2018	Randomized controlled trial	To study the effect of aspirin on cardiovascular events and bleeding in the healthy elderly	Aspirin resulted in a significantly higher risk of major hemorrhage and did not lower cardiovascular disease risk.
Ando et al. [22].	2019	Cohort study	To study the influence of blood pressure on the effects of low dose aspirin in elderly patients with multiple atherosclerotic risks	Low-dose aspirin therapy was associated with an elevated risk of hemorrhagic events.
García Rodri’guez et al. [23].	2020	Cohort study	Bleeding associated with low dose aspirin	Low-dose aspirin is associated with intracranial bleed

Limitations

This review article was subjected to limitations. There was limited availability of studies that focussed on the combined use of aspirin and caffeine in the elderly. Pre-existing systemic conditions in older individuals were not considered. Substantial risk factors for stroke were not considered. The dose at which both caffeine and aspirin could lead to the adverse effect of IC bleed on the brain should be construed. Articles in languages other than English could not be included.

## Conclusions

We conducted this systematic review to establish the association between the two most commonly used substances, caffeine, and aspirin, in causing ICH. Since the mortality of ICH is soaring, it was essential for us to emphasize the effects of caffeine and aspirin on the older populace. This review article explained the mechanism of caffeine and aspirin and how each was independently associated with a risk of intracranial hemorrhage. Based on the reviewed studies, we found that caffeine increased blood pressure and the risk of ICH, which was also seen with medications that contained caffeine. The studies also showed that the prophylactic use of low-dose aspirin was associated with ICH. This literature would be significant as it emphasizes to be prudent on prescribing aspirin to the older classes who are chronic caffeine intakers. It is recommended that future studies should aim to focus on the combined effects of aspirin and caffeine on the brain due to the inadequate availability of information.
